# Mitochondrial Quality Control in Aging and Heart Failure: Influence of Ketone Bodies and Mitofusin-Stabilizing Peptides

**DOI:** 10.3389/fphys.2019.00382

**Published:** 2019-04-10

**Authors:** Phung N. Thai, Lea K. Seidlmayer, Charles Miller, Maura Ferrero, Gerald W. Dorn, Saul Schaefer, Donald M. Bers, Elena N. Dedkova

**Affiliations:** ^1^ Division of Cardiovascular Medicine, Department of Internal Medicine, University of California, Davis, Davis, CA, United States; ^2^ Division of Cardiology, Department of Internal Medicine, University Hospital Würzburg, Würzburg, Germany; ^3^ Comprehensive Heart Failure Center, University of Würzburg, Würzburg, Germany; ^4^ Department of Pharmacology, School of Medicine, University of California, Davis, Davis, CA, United States; ^5^ Department of Internal Medicine, Center for Pharmacogenomics, Washington University School of Medicine, St. Louis, MO, United States; ^6^ Department of Veterans Affairs, Northern California Health Care System, Mather, CA, United States

**Keywords:** heart failure, aging, ketone bodies, β-hydroxybutyrate, mitophagy, mitofusin, Parkin, mitochondrial quality control

## Abstract

**Aim:** Aging and heart failure (HF) are each characterized by increased mitochondrial damage, which may contribute to further cardiac dysfunction. Mitophagy in response to mitochondrial damage can improve cardiovascular health. HF is also characterized by increased formation and consumption of ketone bodies (KBs), which may activate mitophagy and provide an endogenous mechanism to limit the adverse effects of mitochondrial damage. However, the role of KBs in activation of mitophagy in aging and HF has not been evaluated.

**Methods:** We assessed mitophagy by measuring mitochondrial Parkin accumulation and LC3-mediated autophagosome formation in cardiomyocytes from young (2.5 months), aged (2.5 years), and aged rabbits with HF (2.5 years) induced by aortic insufficiency and stenosis. Levels of reactive oxygen species (ROS) generation and redox balance were monitored using genetically encoded sensors ORP1-roGFP2 and GRX1-roGFP2, targeted to mitochondrial or cytosolic compartments, respectively.

**Results:** Young rabbits exhibited limited mitochondrial Parkin accumulation with small (~1 μm^2^) puncta. Those small Parkin puncta increased four-fold in aged rabbit hearts, accompanied by elevated LC3-mediated autophagosome formation. HF hearts exhibited fewer small puncta, but many very large Parkin-rich regions (4–5 μm^2^) with completely depolarized mitochondria. Parkin protein expression was barely detectable in young animals and was much higher in aged and maximal in HF hearts. Expression of mitofusin 2 (MFN2) and dynamin-related protein 1 (DRP1) was reduced by almost 50% in HF, consistent with improper fusion-fission, contributing to mitochondrial Parkin build-up. The KB β-hydroxybutyrate (β-OHB) enhanced mitophagy in young and aging myocytes, but not in HF where β-OHB further increased the number of cells with giant Parkin-rich regions. This β-OHB effect on Parkin-rich areas was prevented by cell-permeable TAT-MP1^Gly^ peptide (thought to promote MFN2-dependent fusion). Basal levels of mitochondrial ROS were highest in HF, while cytosolic ROS was highest in aged compared to HF myocytes, suggesting that cytosolic ROS promotes Parkin recruitment to the mitochondria.

**Conclusion:** We conclude that elevated KB levels were beneficial for mitochondrial repair in the aging heart. However, an impaired MFN2-DRP1-mediated fusion-fission process in HF reduced this benefit, as well as Parkin degradation and mitophagic signaling cascade.

## Introduction

The incidence of cardiovascular disease, including heart failure (HF), has increased steadily in the United States as the general population has aged (Roger et al., 2004; Savarese and Lund, 2017). Mitochondria are essential for energy generation to maintain cardiac contraction during each heartbeat. However, mitochondrial function declines during aging and HF, leading to accumulation of depolarized mitochondria and a decline in ATP production, thereby resulting in impaired ability of the heart to pump blood (Wallace, 2005; Lopez-Otin et al., 2013; Abdellatif et al., 2018). To counteract these adverse effects, cells are able to monitor, mark, and effectively remove damaged mitochondria through a selective mitochondrial macro-autophagy, or mitophagy (Kim et al., 2007; Hammerling and Gustafsson, 2014; Kotiadis et al., 2014; Linton et al., 2015).

Effective degradation and removal of dysfunctional mitochondria is extremely important for cardiac myocyte survival (Linton et al., 2015). Mitochondrial membrane depolarization activates serine/threonine kinase PTEN-inducible kinase 1 (PINK1, encoded by *PARK6*) accumulation on the mitochondrial surface which in turn recruits the E3 ubiquitin ligase (Parkin, encoded by *PARK2*) to the outer mitochondrial membrane from the cytosol (Pickrell and Youle, 2015). Parkin then polyubiquitinates mitochondrial membrane proteins including the mitochondrial outer membrane GTPase mitofusin 2 (MFN2), thereby marking mitochondria for degradation and permitting recognition and binding by the adaptor protein SQSTM1/p62 (Pankiv et al., 2007). SQSTM1/p62 binds the microtubule-associated protein light chain 3 (LC3) on the autophagosome and the ubiquitinated mitochondrial proteins, which tethers the autophagosome to the mitochondrion (Kubli et al., 2015; Moyzis et al., 2015). The autophagosome then surrounds the mitochondrion and fuses with lysosomes, thereby leading to degradation of mitochondrial proteins by lysosomal hydrolases and amino acids recycling (Moyzis et al., 2015).

The level of mitophagy has been shown to be affected by metabolic substrates and activated during nutrient deprivation (i.e., starvation) (He and Klionsky, 2009). In the absence of energy substrates during starvation, there is a shift in metabolism toward increased generation of ketone bodies (KBs), acetoacetate, and β-hydroxybutyrate (β-OHB) in liver mitochondria. Increased formation of KBs is also observed in HF; however, it is unclear whether this KB increase is beneficial for the heart or actually contributes to HF development (Kupari et al., 1995; Lommi et al., 1996, 1997; Aubert et al., 2016; Bedi et al., 2016; Kolwicz et al., 2016). Recent studies (Aubert et al., 2016; Bedi et al., 2016; Huynh, 2016; Kolwicz et al., 2016) suggested that KBs are elevated as an adaptive emergency mechanism to compensate for the reduced free fatty acid oxidation in HF. However, prior studies (Kupari et al., 1995; Lommi et al., 1996, 1997) and recent retrospective analysis of data from 171 to 405 patients admitted to the hospital (Obokata et al., 2017; Arima et al., 2019) reported a direct correlation between an increase in circulating levels of KBs and the decline in cardiac function. KBs might be beneficial in HF *via* their ability to decrease oxidative stress (Shimazu et al., 2013) or serve as an alternative energy source (Veech et al., 2017). While both these processes can affect mitophagy, the role of KBs in regulation of mitochondrial quality control in HF has not been evaluated yet.

Here, we investigated the effect of β-OHB on the basal level of mitophagy in cardiac myocytes from young, aged control, and aged HF rabbits. We found a very low level of basal mitophagy in healthy young rabbit hearts: Parkin accumulation and LC3-mediated autophagosome formation were barely detectable using high-resolution confocal microscopy and western blot analysis. However, mitochondrial uncoupling with the protonophore carbonyl cyanide 4-(trifluoromethoxy)-phenylhydrazone (FCCP) induced a dose-dependent increase in mitochondrial Parkin accumulation and LC3-mediated autophagosome formation. This FCCP-induced level of mitophagy was comparable to that observed in aging cardiac myocytes without FCCP, consistent with upregulation of mitophagy during aging, potentially due to higher mitochondrial uncoupling and/or oxidative stress during aging. Indeed, using oxidative stress and redox balance sensors targeted to cytosolic and mitochondrial compartments (cyto/mito-roGFP2-ORP1 and cyto/mito-roGFP2-GRX1), we found that mitochondrial H_2_O_2_ levels were moderately elevated during aging and reached the highest level in HF. This significant elevation in H_2_O_2_ levels was accompanied by impaired mitophagy in HF myocytes. The enhanced Parkin accumulation in HF myocytes was not matched by increased LC3-I to LC3-II conversion (indication of autophagosome formation). Instead, we observed accumulation of giant Parkin-rich areas in HF, suggestive of mitophagic arrest in HF.

Cell exposure to β-OHB promoted Parkin puncta and LC3-mediated autophagosome formation in young and aging hearts, but not in HF. Despite the fact that Parkin expression was highly elevated, the amount of LC3-mediated autophagosome was actually decreased under conditions of impaired fusion-fission process (both MFN2 and DRP1 expression were decreased). Cell-permeable peptides, which promoted fusion/fission cycling by modulating MFN2 conformation, abolished large Parkin-rich regions formation in HF myocytes. Elevated KBs were beneficial for mitochondrial repair in the aging heart, but that in HF, the impaired MFN2/DRP1-mediated fusion-fission limited benefits from this effect and impaired mitophagic signaling pathway. Together, these data suggest that KB β-OHB enhanced mitophagy flux by restoring oxidative-redox balance in young and aged cardiac myocytes with intact MFN2/DRP1 function, but not in HF where the fusion-fission process is impaired. MFN2-stabilizing peptides could be beneficial in HF, in part by improving mitophagy signaling cascade in HF myocytes.

## Materials and Methods

### Animal Model

All animal handling and laboratory procedures were performed in accordance with the approved protocols of the Institutional Animal Care and Use Committee of the University of California, Davis conforming to the Guide for the Care and Use of Laboratory Animals published by the US National Institute of Health (8th Edition, 2011).

### Rabbit Model of Aging and Heart Failure

Chronic HF was induced in young rabbits by combined volume- and pressure overload resulting from aortic valve insufficiency (AI) and aortic constriction (Pogwizd, 1995; Dedkova et al., 2013; Hegyi et al., 2018). Aortic valve insufficiency was induced by inserting a beveled 5-Fr micropuncture introducer *via* the left carotid artery and pushing it abruptly through the aortic valve several times. Two-dimensional echocardiography was used to monitor the severity of aortic valve damage. Animals were allowed to rest for 2 weeks, and then the second surgery was performed to induce aortic constriction. In this case, the abdominal aorta was tightened with a 4/0-silk ligature, which led to a reduction of aortic diameter of ~25–40% (depending on the severity of AI). Every rabbit underwent echocardiography examination before AI surgery to record baseline parameters and at 2- to 4-week intervals after surgeries. After the initial compensatory hypertrophy, HF developed slowly over the period of ~8–12 months. Rabbits were used for experimentation at a time when signs of HF were established as evident by almost double heart-to-body weight (5.52 ± 0.71 in HF vs. 2.63 ± 0.15 g/ kg in control, *p* < 0.01), increased left ventricular end diastolic diameter (by 34%; *p* < 0.01), decreased fractional shortening (by 23%; *p* < 0.05), and exhibited abdominal ascites and evidence of lung edema (increased lung weight-to-body weight ratio) (Hegyi et al., 2018).

### Cell Isolation

For cardiomyocyte isolation, rabbits were subjected to general anesthesia induced with 2 mg/kg propofol followed by 2–5% isoflurane in 100% oxygen (Hegyi et al., 2018). Then, thoracotomy was performed and the heart was quickly excised and rinsed in cold nominally Ca^2+^-free minimal essential medium (MEM). The right atrium was removed and the aorta opened to visualize the left coronary ostium, which was then cannulated using a 5-F Judkins right catheter (Performa; Merit Medical Systems). Perfusion of the left ventricle and left atrium was established before removal of the right ventricular free wall and application of a purse-string suture to secure the catheter in place. Then, the heart was perfused with MEM solution containing 20 μmol/L Ca^2+^ and 22.5 μg/mg Liberase TH at 37°C until tissue was digested. The left ventricle was cut from the heart and minced, filtered, and washed in a MEM solution containing 50 μmol/L Ca^2+^ and 10 mg/ml bovine serum albumin. Isolated cells were kept in MEM solution with 50 μmol/L Ca^2+^ at room temperature (22–24°C) until used for experimentation or placed in a short-term culture (see below). Cells isolated from healthy age-matched and young rabbits were used for control experiments.

### Confocal Microscopy

Laser scanning confocal microscopy (Nikon A1) was used to follow the changes in mitophagy, mitochondrial membrane potential (ΔΨ_m_), and oxidative and redox status using specific adenoviral constructs and fluorescent indicators as described below.

### Mitophagy Measurements

mCherry-Parkin and GFP-LC3 were expressed *via* adenoviral gene transfer in isolated myocytes that were kept in short-term culture (24–48 h; multiplicity of infection of 500) and used to monitor Parkin accumulation in mitochondria and LC3-mediated autophagosome formation (Kubli et al., 2015). Freshly isolated myocytes were plated on sterile, laminin-coated glass coverslips in PC-1 medium, supplemented with penicillin and streptomycin (50 μg/ml). mCherry-Parkin fluorescence was excited at 540 nm and recorded at 590 nm while GFP-LC3 fluorescence was measured with *λ*_ex_ = 488 nm and *λ*_em_ = 510 nm. Mitophagy signaling cascade was examined by Western blot analysis of PINK1, Parkin, MFN2, DRP-1, SQSTM1/p62, pro-LC3, and LC3-I/LC3-II ratio in cell lysates from young, aged, and HF myocytes. Conformation state of MFN2 was manipulated by cell-permeable engineered mini-peptides to destabilize the fusion-constrained conformation of mitofusin (TAT-MP2^Gly^) and promote the fusion-permissive conformation (TAT-MP1^Gly^), which is shown to reverse mitochondrial abnormalities in cultured fibroblasts (Franco et al., 2016).

### Oxidative Stress and Redox Potential Measurement

To examine oxidative stress and redox balance under basal conditions in young, aged, and HF myocytes, we used adenoviral constructs that allow to express redox-sensitive green fluorescent protein 2 (roGFP2) fused to specific sensor domains to measure changes in either H_2_O_2_ or oxidated glutathione (GSSG) relative to changes in reduced glutathione (GSH) (GSSG/GSH) in mitochondria and cytosol of intact cells (Dey et al., 2016). In roGFP2, two surface-exposed cysteines were introduced into the β-barrel (β-sheet reach region) of standard GFP at positions 147 and 204 (Meyer and Dick, 2010), which mediates redox sensitivity of the construct. roGFP2 has two different and redox-dependent excitation peaks at 405 and 488 nm, and emits fluorescence with maximum at 510 nm (Meyer and Dick, 2010). Oxidation of cysteine leads to the increase in 405 nm excitation peak and corresponding decrease in 488 nm excitation peak while cysteine reduction induces inverse effect. Thus, the ratio between emission at 510 nm and excitation either at 405 or 488 nm can be used as an indicator of the relative amount of oxidized/reduced roGFP2. This ratio reflects the redox state of the cell or cellular compartment in which roGFP is present, with the ratios for the two excitation maxima shifting in opposing directions depending on reduction or oxidation (Meyer and Dick, 2010; Morgan et al., 2011). To specifically detect H_2_O_2_, the redox-sensitive roGFP2 was fused with the yeast peroxidase ORP1 (Gutscher et al., 2009). When ORP1 reacts with H_2_O_2_, it forms a cysteine sulfenic acid (Cys-SOH) in the active site, leading to the formation of protein disulfide bonds in roGFP2, thus shifting the 405/488-nm excitation ratio (Gutscher et al., 2009). Similarly, a fusion construct roGFP2-GRX1 uses human glutaredoxin to sense the changes in oxidized glutathione (GSSG) relative to the level of reduced glutathione (GSH), GSSG/GSH (Dickinson et al., 2010; Meyer and Dick, 2010; Morgan et al., 2011). To calibrate the signal, diamide was added at the end of each experiment to oxidize the GSH pool and obtain *R*_max_ for the roGFP2 probe and DTT to obtain *R*_min_. The detailed procedure of construct generation is described in Gutscher et al. (2008, 2009) and Dey et al. (2016). Mitochondrial targeting of the construct was mediated by fusion of roGFP2-ORP1and roGFP2-GRX1 to the mitochondrial targeting sequence of the first 69 amino acids of subunit 9 of the F_0_ ATPase of *Neurospora crassa* (Schwarzlander et al., 2016). Mito/cyto-roGFP2-ORP1 and Mito/cyto-roGFP2-GRX1 were excited with the 405- and 488-nm laser lines, and emission was detected at with a 510 nm.

### Mitochondrial Membrane Potential (ΔΨ_m_) Measurements

Mitochondrial membrane potential (ΔΨ_m_) was monitored in intact cardiomyocytes using the potentiometric probe tetramethylrhodamine methyl ester (TMRM) (*λ*_ex_ = 543 nm and *λ*_em_ = 565–605 nm) (Dedkova and Blatter, 2012; Seidlmayer et al., 2015, 2016). Cardiomyocytes were loaded with 5 nM TMRM for 30 min at 37°C. 5 nM TMRM was present in all solutions during the experiments. In the end of each experiment, 10 μM FCCP and 1 μM oligomycin were added to calibrate the signal. All data were background corrected.

### SDS-PAGE and Western Blotting Analysis

Left ventricular cardiomyocytes were homogenized in ice-cold lysis buffer (50 mM Tris pH 7.5, 1 mM EDTA, 1 mM EGTA, 10% glycerol, 1% triton X-100, 50 mM NaF, 5 mM Na_4_P_2_O_7_, 1 mM Na_3_VO_4_, 1 mM DTT, and protease inhibitor cocktail) and protein content was determined using the BCA method (Thai et al., 2018). Briefly, 30 μg of protein was separated by 4–15% SDS-PAGE and transferred onto 0.2 μm supported nitrocellulose membrane using standard protocols. Blocked membranes were exposed to primary antibodies for PINK1 (Abcam ab23707), Parkin (Santa Cruz sc-32,282), mitofusin 2 (ab56889), DRP1 (Abcam ab56788), SQSTM1/p62 (Abcam ab56416), and LC3 (MLB, M186–3). GAPDH (Abcam ab9483) or VDAC1 (Abcam ab15895) were used as loading controls. Membranes were visualized using the Licor Odyssey infrared imaging system and analyzed with ImageJ software. Band intensities of all samples were normalized to an internal housekeeping protein that was included on each gel.

### Statistical Analysis

All data were analyzed using unpaired *t*-tests or two-way analysis of variance with Tukey’s *post hoc*, using GraphPad Prism version 7.0 (GraphPad Software Inc., San Diego, USA), and *p* < 0.05 was considered statistically significant. All data are reported as mean ± standard error of the mean (SEM).

## Results

### Parkin Accumulation and LC3-Mediated Autophagosome Formation Is Activated in Cardiomyocytes From the Young Rabbit Hearts in Response to the Mitochondrial Stress

**Figure 1:**
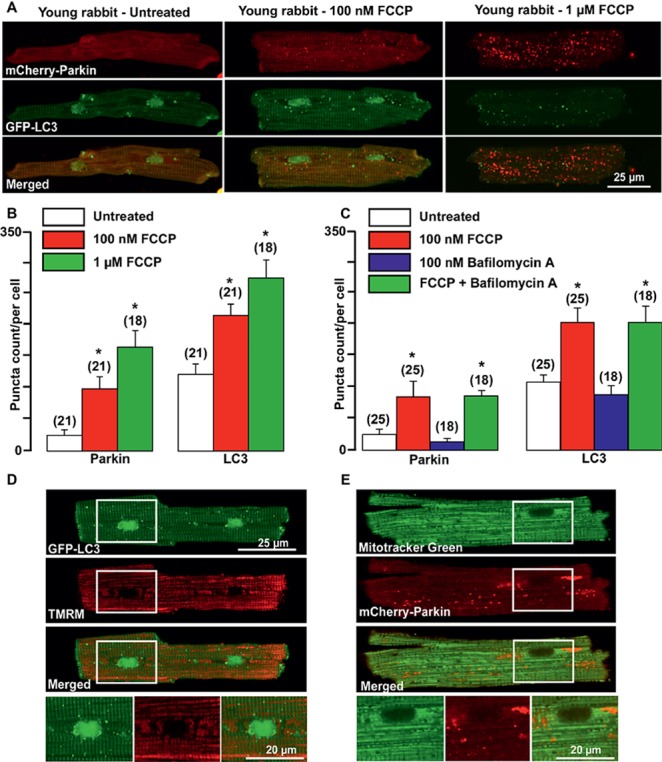
Parkin-mediated mitophagy flux is activated in cardiomyocytes from young rabbit hearts upon treatment with mitochondrial uncoupler FCCP. **(A)** Representative images of mCherry-Parkin and GFP-LC3 distribution in untreated myocytes from young rabbit hearts (left) and cardiomyocytes treated with 100 nM (middle) or 1 μM (right panels) mitochondrial uncoupler FCCP. **(B)** Summary of mCherry-Parkin and GFP-LC3 puncta count per cell in untreated cardiomyocytes from young rabbit and myocytes treated with 100 nM or 1 μM FCCP, respectively. **(C)** In separate experiments, cells were pre-treated with 100 nM Bafilomycin A, a vacuolar H^+^-ATPase inhibitor that prevents autophagosome and lysosome fusion, and then treated with 100 nM FCCP to induce mitophagy. As shown in the summary data, mCherry-Parkin and GFP-LC3 puncta count per cell was not affected by Bafilomycin A1 significantly, indicating the slow mitophagy flux in cardiomyocytes from the young hearts. **(D)** Representative examples of 100 nM FCCP-treated GFP-LC3-expressing control myocyte co-loaded with mitochondrial membrane potential sensitive dye TMRM (left), and **(E)** mCherry-Parkin-expressing myocyte co-loaded with MitoTracker Green to identify mitochondria (right). The images below represent enlarged areas from the region of interest as shown in the rectangular area. Data expressed as mean ± SEM. *n* = number of cells from three different animals per experimental group. *p < 0.05 in treated vs. untreated cells in each group.

It has been demonstrated that damaged mitochondria are removed by selective mitophagy, mediated by the mitochondrial kinase PINK1 and the cytosolic ubiquitin ligase Parkin with subsequent LC3-mediated autophagosome formation and elimination of dysfunctional mitochondria by lysosomes (Moyzis et al., 2015). However, involvement of PINK-Parkin pathway in mitochondrial repair under basal conditions has been questioned (Dorn, 2016; Kubli et al., 2015). We, therefore, examined whether Parkin could be translocated from the cytosol into mitochondria upon mild and severe mitochondrial stress induced by cardiomyocytes exposed to the mitochondrial uncoupler FCCP. As shown in the left panels of Figure 1A, under basal (unstimulated) conditions, a very small amount of mCherry-Parkin puncta was detected in cardiomyocytes from healthy young rabbits of 2.5 months of age with the majority of mCherry-Parkin fluorescence homogeneously distributed in the cytosol. However, when myocytes were exposed to mild or severe mitochondrial stress induced by overnight cell exposure to either 100 nM (middle) or 1 μM (right panels) FCCP, a significant increase in puncta-like mitochondrial Parkin signal was detected. Autophagosome formation was monitored by the appearance of LC3-mediated puncta-like green signal. As shown in Figure 1A, puncta-like green signal was detected under basal conditions and the number of LC3-GFP puncta was gradually increased upon FCCP exposure. As summarized in Figure 1B, both Parkin translocation into the mitochondria and LC3-mediated autophagosome formation increased proportionally with the severity of mitochondrial stress. Cell treatment with 100 nM Bafilomycin A1, a vacuolar H^+^-ATPase inhibitor that prevents fusion of autophagosome with lysosome (Yamamoto et al., 1998), did not affect basal and FCCP (100 nM)-induced Parkin and LC3 puncta count significantly, indicating low mitophagy flux in the young heart (Figure 1C). Mild depolarization with 100 nM FCCP affected mainly mitochondria in perinuclear regions (as monitored with mitochondrial membrane potential-sensitive dye TMRM, Figure 1D), where the majority of LC3-mediated puncta was found in close proximity to depolarized mitochondria. Furthermore, co-staining of mCherry-Parkin-expressing cells with MitoTracker Green revealed that indeed Parkin puncta was accumulated in mitochondrial areas, presumably on the outer mitochondrial membrane (Figure 1E). These data indicate that Parkin-mediated mitophagy exists in healthy young rabbit hearts and Parkin is ready to be recruited into mitochondria even upon mild stress induction.

### Parkin-Mediated Mitophagy Cascade Is Elevated in Aging Hearts but Severely Impaired in Heart Failure

**Figure 2:**
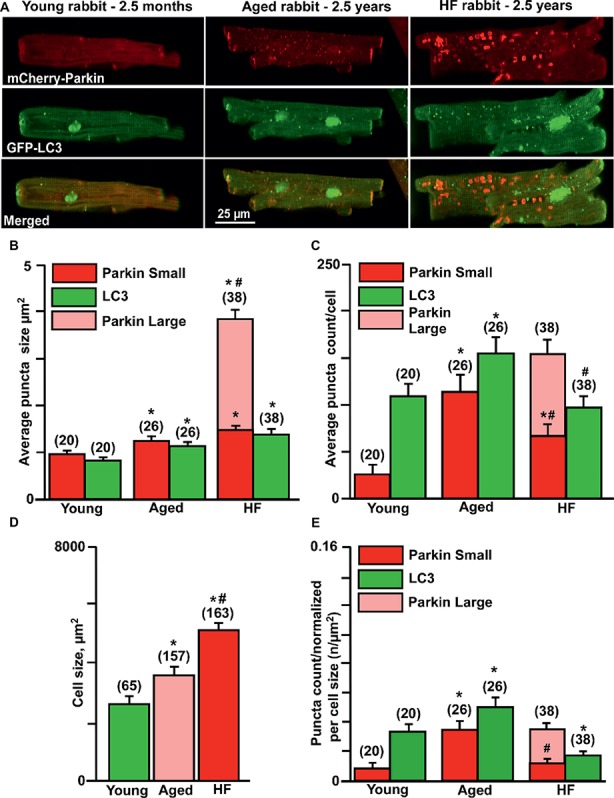
Parkin-mediated mitophagy flux is significantly elevated in aging hearts but impaired in HF. **(A)** Representative images of mCherry-Parkin and GFP-LC3 puncta distribution in young, aged control and HF myocytes. **(B)** Average puncta size in young, aged and HF myocytes. **(C)** Average puncta count per cell in young, aged and HF myocytes. **(D)** Cell size differences in young, aged and HF myocytes. **(E)** Puncta count normalized per cell size in young, aged and aged HF myocytes. Data expressed as mean ± SEM. *n* = number of cells from four young, five aged and six HF animals. *p < 0.05 reflects the difference in aged and HF myocytes vs. young; #*p* < 0.05 aged vs. HF myocytes.

Here, we compared the levels of mitochondrial Parkin accumulation and LC3-mediated autophagosome formation under basal (untreated) conditions in cardiomyocytes from young (2.5 months), aged (2.5 years), and aged rabbits (2.5 years) with HF induced by combined aortic insufficiency and stenosis. Figure 2A shows representative images of cardiomyocytes expressed with mCherry-Parkin (red, upper panels) and GFP-LC3 (green, middle panels), and overlay of both images (merged, lower panels) from three experimental groups. We quantified the signal by counting the number and measuring the size of mCherry-Parkin and GFP-LC3 puncta in individual cells. We determined that the average puncta size in young and aged control cardiomyocytes varied between ~1 and 1.4 μm^2^, reflecting the marking and removal of individual mitochondria (Figure 2B). In HF myocytes, however, we observed formation of very large Parkin-rich regions with an average size 4–5 μm^2^, which was not associated with the increase in LC3-puncta size. In agreement with data presented in Figure 1, very low levels of mitochondrial Parkin accumulation were detected in cardiac myocytes from the young hearts; however, cardiac myocytes from the aged hearts exhibited an almost four-fold increase in Parkin puncta accumulation with a correspondent increase in LC3-mediated autophagosome formation, suggesting upregulation of mitophagy in the aging heart (Figure 2C). By contrast, failing hearts of similar age exhibited a decrease in small Parkin and LC3 puncta and a significant increase in large Parkin-rich regions (Figure 2C, pink bar). Since cell size was increased in both aged control and aged HF myocytes (Figure 2D) due to hypertrophy, we normalized the puncta count per cell size. The normalized data are shown in Figure 2E and demonstrate that Parkin-mediated mitophagy signaling was significantly increased in control cardiac myocytes from the aged hearts, but impaired in aged HF myocytes. The increase in Parkin-rich areas in HF myocytes did not correspond to LC3-mediated autophagosome formation, suggesting that the mitophagy signaling pathway was “jammed” after Parkin recruitment to the mitochondria.

**Figure 3:**
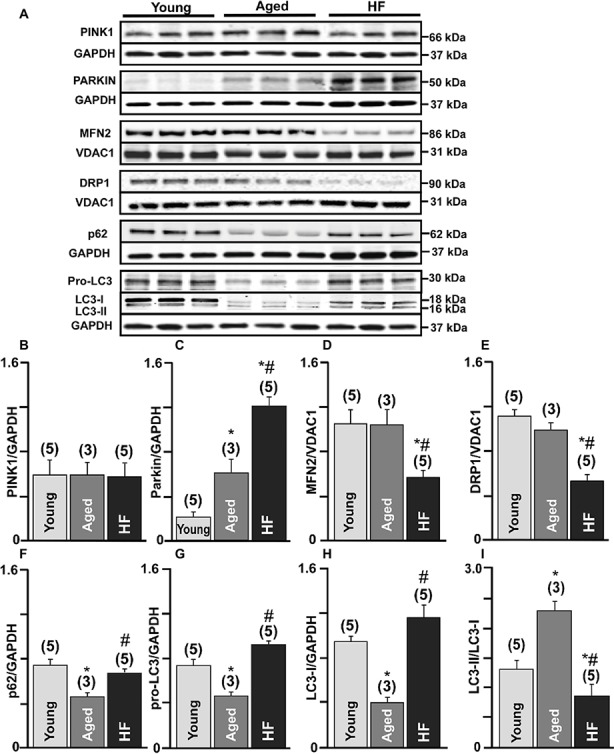
Mitophagy signaling pathway was upregulated in the aging heart but severely impaired in HF. **(A)** Expression of the proteins in PINK-Parkin mitophagy pathway in cardiac myocytes from young, aged and failing hearts: PINK-1, Parkin, mitofusin 2 (MFN2), dynamin-related protein 1 (DRP1), SQSTM1/p62, pro-LC3, and LC3-I/LC3-II. GAPDH staining was used to confirm equal protein loading for each experimental group. **(B–H)** Summary analysis of PINK-1, Parkin, mitofusin 2 (MFN2), DRP1, SQSTM1/p62, pro-LC3, LC3-I protein expression normalized to GAPDH or VDAC1 as indicated in the figure. **(I)** Quantification of LC3-I conversion to LC3-II protein expression presented as LC3-II/LC3-I ratio. Data expressed as mean ± SEM. *n* = 3–5 animals per group, *p < 0.05 reflects a difference in aging and HF hearts vs. young hearts, #*p* < 0.05 reflects a difference in HF hearts vs. corresponding age-matched control hearts.

We further confirmed these results by measuring protein expression levels of the essential proteins involved in the mitophagy signaling cascade. No significant changes in PINK-1 expression were observed between the three experimental groups (Figures 3A,B). We detected a very low level of Parkin in control (0.2 ± 0.04 Parkin/GAPDH ratio, *n* = 5 hearts) hearts, three-fold increase in aged hearts (0.60 ± 0.26, *n* = 3 hearts), and the highest expression level in HF (1.2 ± 0.07, *n* = 5 hearts, *p* < 0.01) (Figures 3A,C). However, the expression level of mitofusin 2 (MFN2), which creates a hub for autophagosome formation, was significantly decreased in HF myocytes (56.73 ± 14%, *N* = 5 hearts, *p* < 0.01) compared to nonfailing age-matched myocytes (99.9 ± 15%, *N* = 3) and myocytes from the young hearts (taken as 100%, *N* = 5) (Figures 3A,D). Furthermore, the expression of the dynamin-related protein 1 (DRP1, fission blocker) was significantly decreased in failing hearts compared to the young and corresponding age-matched control rabbits (Figures 3A,E). Expression levels of the adaptor protein SQSTM1/p62 (62 kDa) (Figures 3A,F) and pro-LC3 (30 kDa) (Figures 3A,G) were decreased in aged hearts compared to the young hearts, indicating their degradation *via* autophagy pathway. In HF, however, p62 expression level was not changed significantly, but pro-LC3 level was elevated compared to the young hearts, suggesting an impairment in autophagosome formation. LC3 (microtubule-associated protein 1 light chain 3) is a mammalian autophagosomal marker (an analog of Atg8 in yeasts) which is translated as a full-length 30 kDa precursor (Kabeya et al., 2000). When pro-LC3 is cleaved at the C-terminus, it generates the cytosolic LC3-I (18 kDa) form. Sequentially, LC3-I is converted to LC3-II (16 kDa) that gets recruited to autophagosomal membranes *via* conjugation to phosphatidylethanolamine (Kabeya et al., 2004). The conversion of cytosolic LC3-I to its lipidated membrane-bound form LC3-II indicates autophagosome formation, and the amount of LC3-II typically correlates with the number of autophagosomes (Kabeya et al., 2000, 2004). Western blot analysis of endogenous LC3 levels confirmed a significant decrease in LC3-I (Figures 3A,H) and increase in the LC3-II/LC3-I ratio (Figures 3A,I) in the aging heart but not in HF (Figure 3G). Taken together, these measurements demonstrate that there was an increase in the removal of damaged mitochondria *via* mitophagy in cardiomyocytes from the aging hearts compared to similar age HF myocytes.

### Large Areas With Severely Depolarized Mitochondria Are Observed in Heart Failure Cardiomyocytes

**Figure 4:**
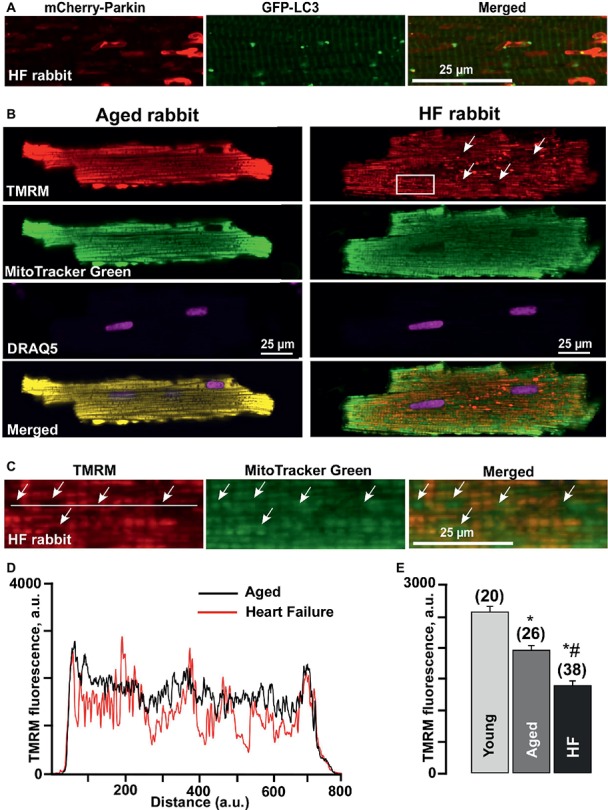
Formation of Parkin-rich areas in HF myocytes was associated with severely depolarized mitochondria. **(A)** Shown are Parkin-rich areas and GFP-LC3 puncta distribution in HF myocytes under the higher magnification. **(B)** Shown are representative images of healthy control aged and HF myocytes loaded with mitochondrial membrane potential sensitive dye TMRM (red), Mitotracker Green (to identify mitochondria), DRAQ5 (to identify nuclei) and overlay of all (merged). **(C)** Magnified images from the rectangular area shown in B in HF myocytes reflect the areas with completely depolarized mitochondria. **(D)** Summary of mean mitochondrial membrane potential in cardiac myocytes from young, aged, and HF myocytes. *n* = number of cells from four young, five aged, and six HF animals. *p < 0.05 in aged and HF vs. young; #*p* < 0.05 reflects a difference in HF hearts vs. corresponding age-matched control hearts. **(E)** Plot profile summaries of TMRM fluorescence obtained from the lines placed across the cells in aged and HF myocytes.

A detailed look at the Parkin-rich areas in HF myocytes (Figure 4A) revealed that these areas could represent a fusion of several mitochondria (approximately 4–5 mitochondria determined by size), which were marked for degradation but did not get removed *via* mitophagy. We, therefore, estimated changes in the mitochondrial membrane potential (ΔΨ_m_) in cardiomyocytes loaded with the membrane potential-sensitive dye TMRM and determined that cardiomyocytes from failing hearts exhibited large areas with severely depolarized mitochondria (seen as a dark spots in Figure 4B). By contrast, TMRM fluorescence demonstrated a typical mitochondrial pattern in similar age nonfailing cardiomyocytes, as evident by the appearance of regular blocks with TMRM-loaded mitochondria. We co-stained the cells with mitochondrial marker MitoTracker Green and determined that these giant areas with decreased mitochondrial membrane potential indeed contained mitochondria (see rectangular inset in Figure 4B and zoomed images in Figure 4C). Plot profiles of the TMRM fluorescence obtained over the line placed across either aged or HF cells showed the areas in HF myocytes where the mitochondrial membrane potential dropped ~75% (Figure 4D). Furthermore, the average level of TMRM fluorescence collected from the whole cell was decreased from 2,586 ± 89 a.u. (*n* = 20) in mitochondria of young cardiomyocytes to 1,967 ± 81 a.u. (*n* = 26, *p* < 0.05) in aged and to 1,404 ± 72 a.u. (*n* = 38, *p* < 0.05) in HF myocytes (Figure 4E), suggesting that enhanced Parkin accumulation in aged and HF myocytes was in response to the mitochondrial membrane depolarization observed under basal conditions in aged and HF myocytes.

### Effects of Ketone Bodies on Oxidative Stress and Redox Potential in Young, Aged, and Failing Heart

When mitochondria utilize oxidative phosphorylation to convert dietary substrates such as proteins, carbohydrates, and fat into ATP, they also generate small amounts of reactive oxygen species (ROS) as a by-product (Jezek et al., 2018). Under physiological conditions, low levels of ROS activate various signaling mechanisms that in turn upregulate the antioxidant defense system and autophagy required to protect cells against oxidative stress-associated damage. However, higher ROS levels can limit mitophagy, leading to accumulation of damaged mitochondria and compromising cell viability and function (Jezek et al., 2018). To examine whether a difference in oxidative stress and redox balance plays a role in mitophagy activation in aged and HF myocytes, we used adenoviral constructs which allow expression of redox-sensitive GFP2 (roGFP2) fused to specific sensor domains to monitor changes in H_2_O_2_ (oxidant) or glutathione redox potential (E_GSH_) in mitochondria and cytosol of intact cells (Gutscher et al., 2008; Dey et al., 2016). To measure H_2_O_2_, the redox-sensitive roGFP2 was fused with the yeast peroxidase ORP1 (Gutscher et al., 2009), and targeted either to cytosolic or mitochondrial compartment. Redox potential was monitored in cells expressing roGFP2-GRX1 that utilizes human glutaredoxin-1 which specifically catalyzes the equilibration between the redox pair of interest, reduced glutathione (GSH) and oxidized glutathione (GSSG), and the reporting redox couple (roGFP2_red_ and roGFP2_ox_) (Gutscher et al., 2008; Dickinson et al., 2010; Meyer and Dick, 2010; Morgan et al., 2011). Cells protect themselves from nonphysiological ROS concentrations by employing antioxidant molecules which utilize thiol groups to catalytically detoxify themselves from free radicals. Glutathione is the most abundant antioxidant molecule employed by the cell (Ribas et al., 2014). GSH reduces ROS through the glutathione-peroxidase enzyme, producing water and oxidized GSH (GSSG), which is regenerated back to GSH by glutathione reductase at the expense of NADPH (Morgan et al., 2011).

**Figure 5:**
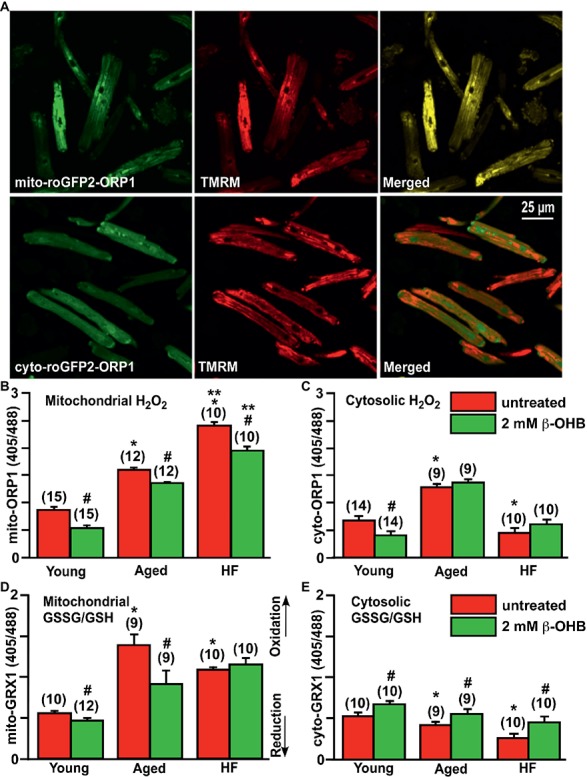
Effect of ketone body (KB) β-hydroxybutyrate (β-OHB) on mitochondrial and cytosolic oxidative stress and redox balance in young, aging and failing hearts. **(A)** Shown are representative images of untreated young rabbit cardiomyocytes expressing mito-roGFP2-ORP1 (green, upper left panel) or cyto-roGFP2-ORP1 (green, lower left panel) and same cells loaded with mitochondrial membrane potential sensitive dye TMRM to identify mitochondria (red, middle panels), and overlay of both channels (merged, right panels). **(B)** Mitochondrial basal H_2_O_2_ levels were the highest in HF myocytes compared to both young and aged cardiomyocytes. Cell treatment with 2 mM β-OHB significantly decreased H_2_O_2_ levels in all experimental groups. **(C)** Cytosolic H_2_O_2_ levels were the highest in aged cardiomyocytes compared to both young and HF myocytes. Cell treatment with 2 mM β-OHB decreased cytosolic H_2_O_2_ levels in young hearts, but did not change the oxidative stress in aged and HF myocytes. **(D)** Levels of the mitochondrial oxidized to reduced glutathione (GSSG/GSH) were the highest in the aging heart and elevated in HF compared to the young hearts. 2 mM β-OHB shifted the mitochondrial GSSG/GSH ratio toward reduced glutathione in both young and aging myocytes but not in HF myocytes. **(E)** The level of the cytosolic GSSG/GSH was progressively declining during aging and HF, and partially increased by 2 mM β-OHB treatment in all experimental groups. Data expressed as mean ± SEM. *n* = number of cells from three different animals per each group. Red bars represent untreated cells; green bars represent cells treated with 2 mM β-OHB for 24 h. *p < 0.05 reflects a significance in aged and HF rabbits vs. young rabbits; **p < 0.05 between HF and HF + OHB vs. aged and aged + OHB groups, #*p* < 0.05 reflects a significance in 2 mM β-OHB-treated cells vs. corresponding untreated groups.

As shown in Figure 5 (top panels), mitochondrial localization of mito-roGFP2-ORP1 (green signal at *λ*_ex_ = 488 nm) was verified by co-staining with the mitochondrial membrane potential sensitive dye TMRM (red signal). As evident in almost complete overlay of the both signals (Figure 5A, top panels, merged), mito-roGFP2-ORP1 was located in mitochondria while the cyto-roGFP2-ORP1 originated primarily from cytoplasm (Figure 5A, bottom panels, merged). Similar localization was observed with the GRX1-roGFP2 probes targeted to the mitochondria (mito-roGFP2-GRX1) and cytosol (cyto-roGFP2-GRX1).

Using these ratiometric fluorescent sensors, we detected that basal level of mitochondrial H_2_O_2_ was progressively increased in aged myocytes compared to cardiac myocytes from young rabbits, and in HF myocytes compared to aged myocytes (Figure 5B). Interestingly, the basal level of cytosolic H_2_O_2_ was found to be the highest in aged hearts, and lowest in HF myocytes compared to the young heart (Figure 5C). As noted above, it has been suggested that KBs (and specifically β-OHB) could provide a beneficial effect *via* suppression of oxidative stress (Shimazu et al., 2013). Therefore, we treated cells with 2 mM β-OHB for 24 h, a concentration which is close to that reported in rabbit HF (Miller et al., 2018), and determined that β-OHB significantly decreased mitochondrial levels of H_2_O_2_ in all experimental groups (Figure 5B). Cytosolic levels of H_2_O_2_, however, were only decreased in control myocytes by β-OHB but did not change significantly in aged and HF hearts (Figure 5C). When we evaluated glutathione redox status in all experimental groups, we found that mitochondrial GSSG/GSH ratio was highest in the aging heart, and also elevated in HF compared to the young heart (Figure 5D). The mitochondrial GSSG/GSH ratio was most likely in response to the elevated H_2_O_2_ as detected with mito-roGFP2-ORP1 (see Figure 5B). Cell treatment with 2 mM β-OHB decreased GSSG/GSH ratio in mitochondria of young and aged myocytes without any effect in HF myocytes. The cytosolic GSSG/GSH ratio, however, progressively declined in aged and HF myocytes compared to myocytes from the young heart. This decline was effectively eliminated by cell treatment with 2 mM β-OHB in all experimental groups (Figure 5E). Together, these data indicate that even under basal conditions (i.e., without additional stress stimulus), mitochondrial ROS levels were moderately elevated in aged myocytes and were significantly higher in HF myocytes compared to young control cardiomyocytes (*p* < 0.05). Accordingly, mitochondrial antioxidant GSH was oxidized to detoxify ROS produced in mitochondria of aging hearts, but to a lesser degree in HF (Figure 5D). Cytosolic GSSG/GSH ratio did not increase in aged myocytes compared to young cardiomyocytes despite the elevated levels of H_2_O_2_. Both basal cytosolic H_2_O_2_ and GSSG/GSH remained low in HF compared to age-matched controls (Figures 5C,E). It is known that changes in GSSG/GSH ratio are important for regulation of ROS-triggered signal transmission, which occurs even under conditions of mild oxidative stress and can trigger physiological cellular responses including PINK1/Parkin-mediated mitophagy (Xiao et al., 2017).

### Ketone Body Enhanced Mitochondrial Quality Control in Young and Aging Hearts but Not in HF Hearts

**Figure 6:**
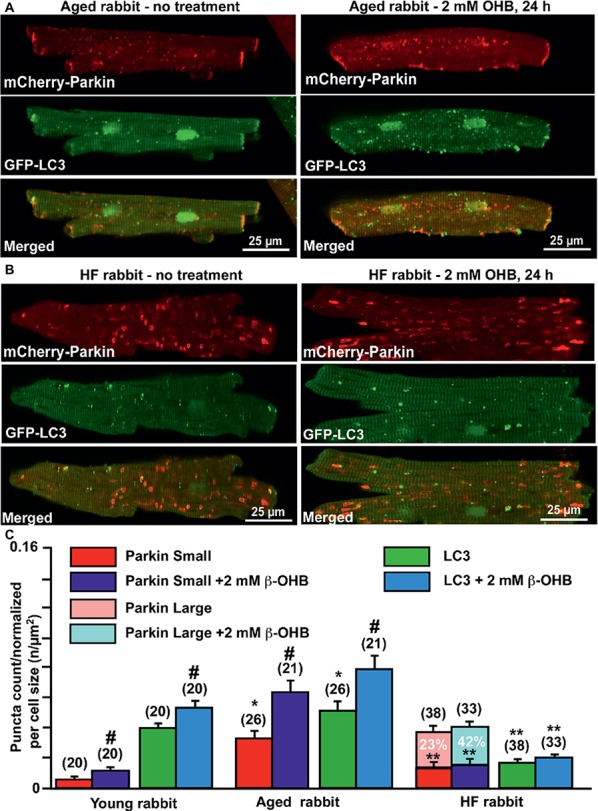
Ketone body β-hydroxybutyrate (β-OHB) enhances mitophagy in young and aging hearts but not in heart failure (HF). **(A)** Shown are representative images of untreated control aged myocytes and myocytes treated with 2 mM β-OHB for 24 h. **(B)** Shown are representative images of untreated HF myocytes and HF myocytes treated with 2 mM β-OHB for 24 h. **(C)** Summary analysis of β-OHB effect on mitochondrial Parkin puncta accumulation and LC3-mediated autophagosome formation in young, aged, and HF myocytes. Data expressed as mean ± SEM. **(C)** Summary analysis of **β-**OHB effect on mitochondrial Parkin accumulation and LC3-mediated autophagosome formation in young, aged, and HF myocytes. Data expressed as mean ± SEM. *n* = number of cells from four young, five aged, and six HF animals. *p < 0.05 reflects a significance in aged and HF rabbits vs. young rabbits; **p < 0.05 reflects a significance in aged vs. HF rabbits, #*p* < 0.05 reflects a significance in 2 mM β-OHB-treated cells vs. corresponding untreated groups.

Since we found significant effects of the KB β-OHB on both ROS and glutathione levels, we next examined whether β-OHB can affect the mitochondrial repair process *via* mitophagy. We performed experiments similar to those described in Figure 2, but this time treated myocytes from young, aging, and HF hearts with 2 mM β-OHB for 24 h. Figures 6A,B show representative images of aged control and HF myocytes with expressed mCherry-Parkin and GFP-LC3 under basal conditions (left panels) and after treatment with 2 mM OHB for 24 h (right panels). From the right panels, it is apparent that cell treatment with 2 mM β-OHB significantly increased the amount of small Parkin puncta (upper panels) and LC3-mediated autophagosome formation (middle panels) in cardiac myocytes from the aging heart. The overlay of both signals indicates the increased formation of LC3-mediated autophagosome upon Parkin recruitment to mitochondria following β-OHB treatment (low panels). In HF conditions after β-OHB treatment, we still observed the presence of large Parkin-rich areas which did not correlate with the increase in LC3-mediated autophagosome formation. As summarized in Figure 6C, 2 mM β-OHB significantly increased both mCherry-Parkin recruitment in mitochondria and LC3-mediated autophagosome formation in both young and aging myocytes but not in HF. The amount of small Parkin puncta was decreased in HF conditions treated with β-OHB compared to the age-matched control, but was not different from untreated HF cells. The number of large Parkin areas, normalized per cell size, did not change in β-OHB-treated HF myocytes compared to untreated HF myocytes; however, the percent of cells showing these large Parkin-rich areas was even higher in β-OHB-treated HF myocytes (42 vs. 23% in untreated) (Figure 6C). We conclude that while 2 mM β-OHB effectively enhanced Parkin recruitment and autophagosome formation in young and aging myocytes, it did not improve mitochondrial repair process in HF.

### Role of Mitofusin 2 in Mitochondrial Quality Control in Aging and Heart Failure

Contact sites between mitochondria and the endoplasmic reticulum (ER) act as crucial signaling hubs in the context of nonselective, starvation-induced autophagy, where they serve as the site of autophagosome formation (Hamasaki et al., 2013; Kishi-Itakura et al., 2014). Indeed, autophagosome biogenesis is impaired in cells with defective mitochondria-ER tethering (Hamasaki et al., 2013), as lipid transfer between organelles may be important for their formation (Hailey et al., 2010; Klecker et al., 2014; Yoshii and Mizushima, 2015). Mitofusin-2 (MFN2) is a mitochondria-ER tether required for starvation-induced autophagosome formation in mammals (De Brito and Scorrano, 2008; Hamasaki et al., 2013; Naon et al., 2016) which is ubiquitinated by Parkin and rapidly turned over by the proteasome (Tanaka et al., 2010). Since we detected an increase in both Parkin expression and formation of giant Parkin-rich areas in HF myocytes, but a decrease in MFN2 expression (Figure 3), we hypothesized that a decrease in MFN2-mediated mito-ER contact sites impairs mitophagy under conditions of enhanced Parkin recruitment, leading to the formation of Parkin-rich areas with dysfunctional mitochondria (see Figures 2, 4).

To test our hypothesis, we treated myocytes from HF hearts and corresponding age-matched control hearts with cell-permeable peptides that can either destabilize the fusion-constrained conformation of mitofusin 2 (TAT-MP2^Gly^) or promote the fusion-permissive conformation (1 μM TAT-MP1^Gly^) (Franco et al., 2016). The fusion-mediated mixing of mitochondrial content was more than doubled by cell treatment with 1 μM TAT-MP1^Gly^ and reversed mitochondrial abnormalities in cultured fibroblasts and neurons that harbor Charcot-Marie-Tooth disease type 2 (CMT2A) while 1 μM TAT-MP2^Gly^ completely suppressed mitochondrial fusion (Franco et al., 2016; Rocha et al., 2018). We, therefore, examined whether promoting or inhibiting fusion with these peptides affect mitochondrial Parkin accumulation and LC3-mediated autophagosome formation in HF myocytes and compared it to their age-matched controls.

**Figure 7:**
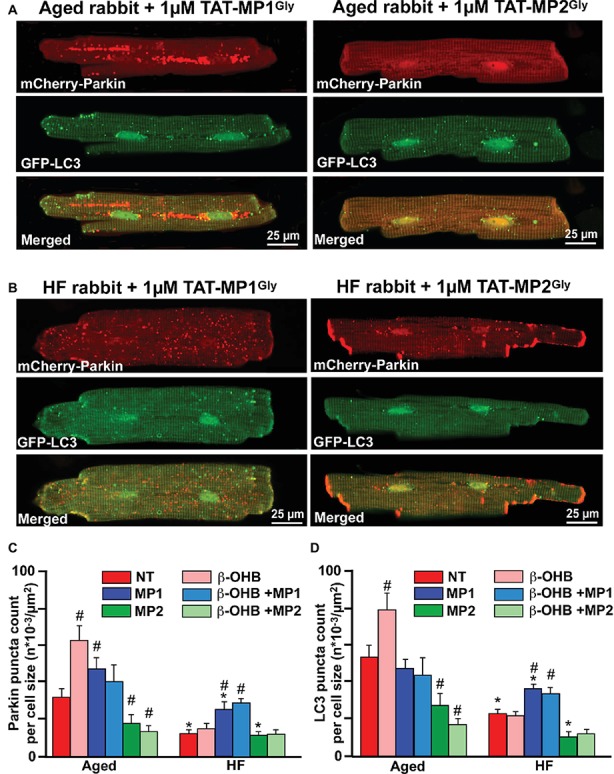
Stabilizing Mfn2 conformation prevents giant fusion formation and improves mitophagy coupling in HF myocytes. **(A)** Representative images showing mCherry-Parkin accumulation and LC3-GFP-mediated autophagosome formation in aged myocytes treated with 1 μM of either TAT-MP1^Gly^ (left) or TAT-MP2^Gly^ (right) peptides. **(B)** Representative images showing mCherry-Parkin accumulation and LC3-GFP-mediated autophagosome formation in HF myocytes treated with 1 μM of either TAT-MP1^Gly^ (left) or TAT-MP2^Gly^ (right) peptides. **(C)** Cell treatment TAT-MP1^Gly^ (MP1) increased Parkin puncta accumulation in aged myocytes and HF cardiomyocytes, while 1 μM TAT-MP2^Gly^ (MP2) decreased mCherry-Parkin puncta accumulation in both aged and HF myocytes. β-OHB did not increase the percentage of cells with Parkin-rich areas in cells treated with MP1. **(D)** Cell treatment TAT-MP1^Gly^ (MP1) did not affect LC3-mediated autophagosome formation in aged myocytes but increased it HF cardiomyocytes. Cell treatment with 1 μM TAT-MP2^Gly^ (MP2) decreased mCherry-Parkin puncta accumulation in both aged and HF myocytes. Data expressed as mean ± SEM. *n* = number of cells from three different animals per each group. *p < 0.05 reflects a significance in HF rabbits vs. aged rabbits; #*p* < 0.05 reflects a significance in peptide-treated cells vs. corresponding untreated groups.

As shown in Figure 7, cells treated with 1 μM TAT-MP1^Gly^ for 24 h, which promoted fusion-mediated content exchange and subcellular trafficking by MFN2, significantly increased both small mCherry-Parkin puncta accumulation and GFP-LC3-mediated autophagosome formation. Most importantly, we did not observe any giant Parkin-rich areas in HF myocyte treated with TAT-MP1^Gly^ (Figure 7B). However, cell treated with 1 μM TAT-MP2^Gly^, which suppressed fusion-mediated content exchange by MFN2 (Franco et al., 2016), led to a decrease in of Parkin puncta and LC3-mediated autophagosome formation in aged control myocytes but did not affect these parameters significantly in HF myocytes, suggesting that mitochondrial content exchange and trafficking was already suppressed in HF. Moreover, when cells were treated with 2 mM β-OHB and 1 μM TAT-MP1^Gly^ simultaneously, we observed further increase in Parkin puncta and LC3-mediated autophagosome formation in HF myocytes, indicating that β-OHB stimulates Parkin translocation in both aging and HF; however, in conditions of impaired MFN2-mediated content exchange and trafficking, β-OHB seems to enhance (Figure 6C) formation of Parkin-rich areas in mitochondria. This suggests that improving MFN2-mediated trafficking and proper fusion in mitochondria could enhance mitochondrial repair and recycling in HF.

## Discussion

In this study, we examined the role of KB β-hydroxybutyrate in the process of the removal and repair of damaged mitochondria, known as mitophagy, in young, aged, and failing hearts. Severely depolarized mitochondria that are unable to maintain proper function are selectively eliminated *via* PINK1-Parkin dependent mitophagy (Gottlieb and Pourpirali, 2016). First, we demonstrated that, when compared to young hearts, the PINK1/Parkin-mediated mitophagy signaling pathway was significantly elevated in cardiomyocytes from the aging hearts, but impaired in HF hearts. The impairment of the mitophagic signaling cascade in HF was characterized by accumulation of large Parkin-rich areas which did not correlate with LC3-mediated autophagosome formation. In fact, the number of small Parkin and LC3 puncta declined in HF compared to a similar age healthy heart (Figure 2). Accumulation of these large Parkin-rich regions corresponded with the areas with completely depolarized mitochondria (Figure 4), indicating that these mitochondria were marked by Parkin for degradation but did not get removed due to impaired mitophagy. In concert with this observation, protein expression analysis of several key proteins involved in the mitophagic signaling cascade revealed that Parkin expression was significantly elevated, while expression of both MFN2 and DRP1 was decreased in the failing heart compared to young and aged hearts (Figure 3). A previous study (Chen and Dorn, 2013) demonstrated appearance of morphologically and functionally abnormal mitochondria in MFN2-deficient mouse cardiomyocytes that caused respiratory dysfunction and dilated cardiomyopathy. However, the precise role of MFN2 in cardiac function (and mitophagy) is still controversial. Some studies have suggested that Parkin inhibits mitochondrial fusion by degrading both MFN1 and MFN2, in order to prevent fusion of healthy and unhealthy (depolarized) mitochondria (Jin and Youle, 2012). Other studies demonstrated that mitofusins promote fusion-mediated mitochondrial content exchange, subcellular trafficking, and serve as a hub for autophagosome formation site (Chen and Dorn, 2013; Shirihai et al., 2015). Indeed, inhibition of the fusion-fission cycle by overexpression of the dominant-negative DRP1 construct which blocks fission, resulted in mitophagy inhibition (Twig et al., 2008). Consistent with this finding, impaired fission in HF myocytes could be secondary to reduced expression level of DRP1 (Figures 3A,E) which then led to the accumulation of large mitochondrial Parkin-positive regions (Figure 2A, right panels). Our data are in agreement with findings from Sadoshima’s lab that demonstrated the critical role of DRP1 in mitochondrial repair *via* autophagy following pressure overload induced by transverse aortic constriction (TAC) in mice (Shirakabe et al., 2016). Their study revealed that DRP1 expression, as well as expression of p62 and LC3-II, was transiently upregulated within 3–12 h following TAC, gradually declined 5 days post-TAC, and reached a minimum expression level 30 days post-TAC. Furthermore, they demonstrated that suppression of mitophagy preceded development of mitochondrial dysfunction and HF, and that restoration of mitophagy protected the heart against HF development (Shirakabe et al., 2016). Cardiospecific knockout of DRP1 exacerbated pressure overload-induced cardiac dysfunction (Shirakabe et al., 2016) and dysfunction following ischemia-reperfusion (Ikeda et al., 2015), emphasizing the important role of DRP1 in regulation of cardiac function. Furthermore, DRP-1 KO cardiac myocytes had increased number of elongated and damaged mitochondria (Ikeda et al., 2015).

Next, we examined whether the inability of MFN2 to form the proper contact sites is the critical step in the observed “clogging” in mitophagical signaling cascade. To test this possibility, we treated cells with peptides which lock MFN2 in specific conformations that either promote or inhibit fusion. As shown in Figure 7, correcting cell fusion with TAT-MP1^Gly^ improved mitophagy flux in HF myocytes. Importantly, we did not observe Parkin-rich areas in HF myocytes treated with TAT-MP1^Gly^. By contrast, cells treated with TAT-MP2^Gly^, which destabilizes mitofusin conformation, decreased the number of small Parkin and LC3-mediated puncta in aged myocytes, but did not have similar effects in HF myocytes. This lack of effect confirms that the fusion-fission process was already impaired in HF myocytes. These data again emphasize the importance of the PINK1-Parkin-MFN2-mediated mitophagic signaling cascade in cardiac myocytes to enable proper mitochondrial repair.

The role of Parkin in aging and healthy hearts is still debated. No significant abnormalities in cardiac function were observed in Parkin knockout (KO) mice which were monitored from the birth until 12 months of age (Kubli et al., 2013b). However, electron microscopy analysis revealed accumulation of large clusters of small, round mitochondria in the hearts of 3-month-old mice, and abnormally shaped mitochondria with a large number of electron-dense granules at 6 months of age (Kubli et al., 2013a). Cell damage in response to ischemia-reperfusion injury was worse in Parkin KO mice compared to a wild-type mice (Kubli et al., 2013b), while ischemia-reperfusion led to upregulation of Parkin in the border infarct area in wild-type animals. These data emphasize the important role of the Parkin-mediated mitophagy pathway for mitochondrial repair and cardioprotection.

Our data presented in Figures 1–3 demonstrate that the PINK-1/Parkin mitophagy pathway is active in young hearts; however, at a very low level compared to the aged hearts, most likely due to low oxidative stress observed in healthy young hearts (Figure 4). Parkin-mediated mitophagy flux could be readily activated in the young heart upon introduction of mild or severe stress by cell treatment with low (100 nM) or high (1 μM) concentrations of the mitochondrial uncoupler FCCP (Figure 1). The level of Parkin puncta accumulation upon introduction of mild stress with 100 nM FCCP in young cardiac myocytes reached the same levels as it was detected in the aging heart at the basal levels (with no additional stress factor as shown in Figure 2). Our data support the findings of Song et al. (2015), where Parkin mRNA and protein expression levels were barely detectable in healthy mouse hearts. This is actually a very important observation since the majority of studies investigating the role of PINK1-Parkin mitophagy pathway were performed in young 3-month-old mice, an age comparable to a 20-year-old human (Flurkey et al., 2007). By contrast, our work was performed in nearly 3-year-old New Zealand rabbits, a more suitable model for studying aging as this age represents a 50- to 60-year-old human. Moreover, rabbits have action potential and calcium handling characteristics which are very close to those observed in the human heart (Pogwizd, 1995; Hegyi et al., 2018). We therefore conclude that the PINK1-Parkin-mediated mitophagy pathway is active in both young and aging hearts under basal conditions but to a greater degree in aging hearts.

We found that the level of mitochondrial oxidative stress was significantly elevated under basal conditions in aged and failing hearts compared to the young hearts (Figure 5). In addition, the elevation in the basal level of oxidative stress in the aging heart was matched by an increase in oxidized glutathione (GSSG) levels relative to the reduced glutathione (GSH) (Figures 5B,D) and upregulation of the mitophagic signaling cascade (Figures 2, 3). To the contrary, basal levels of mitochondrial H_2_O_2_ were significantly higher in HF hearts compared to the aged hearts, while the GSSG/GSH ratio was lower compared to the aging heart, suggesting an impairment in antioxidant defense. Interestingly, the basal level of cytosolic H_2_O_2_ and GSSG/GSH ratio was lower in HF myocytes compared to young and aged control myocytes (Figures 5C,E). Redox homeostasis inside the cell depends on the balance between ROS generation and their scavenging by antioxidant enzymes and molecules such as glutathione (GSH) (Aon et al., 2010; Dey et al., 2016), which can be represented by the ratio of GSSG/GSH (Lu, 2013; Ribas et al., 2014). In the current study, changes in the GSSG/GSH ratio (Figures 5D,E in response to oxidative stress as measured in Figures 5B,C) reflect the redox buffering capacity in mitochondria or cytosol. We found that cells treated with the KB β-OHB at the levels typically observed in HF significantly elevated all steps in the mitophagical signaling cascade in the aging heart but not in HF, despite decreased H_2_O_2_ levels in the mitochondria of all groups (see Figure 5B). Oxidative stress stems from a dysbalance between increased generation of pro-oxidants and the diminished activity of the antioxidant system (Aon et al., 2010). As shown in Figure 5D, the GSSG/GSH ratio was lower in HF myocytes compared to the aged myocytes, and was not affected by β-OHB treatment, indicating that antioxidant defense was compromised in HF conditions. It is, therefore, fair to say that KBs regulate not only cellular bioenergetics and metabolism to facilitate the metabolic switch in HF but also the oxidative stress response (Figure 5) and mitochondrial quality control (Figure 6).

We demonstrated that β-OHB effectively stimulated mitophagy signaling in both young and aging hearts (Figure 6). This is in agreement with findings in neurons where β-OHB stimulated autophagic flux and suppressed cell death induced by glucose deprivation (Camberos-Luna et al., 2016). In HF myocytes, however, KB β-OHB almost doubled the percentage of HF cells with Parkin-rich regions; however, this did not increase LC3-mediated autophagosome formation, suggesting that even though there was upregulation in Parkin, the impairment in mitophagic signaling persisted or was even exacerbated. This effect was prevented by cell treatment with a peptide which stabilizes MFN2 conformation and enhances proper fusion-fission. We, therefore, concluded that β-OHB itself is unlikely to cause clogging of mitophagic flux. Formation of Parkin-rich areas was rather due to improper MFN2/DRP1-mediated fusion-fission process in HF. This is evident by the fact that cell treatment with TAT-MP1^Gly^, which corrects mitochondrial fusion, and may improve the mitophagic process in HF myocytes in the absence and presence of β-OHB (Figures 7C,D).

## Conclusions

Our study demonstrates that in rabbit ventricular myocytes, Parkin-mediated mitophagy exists even in young hearts and Parkin is ready to be recruited to mitochondria in response to a mild mitochondrial stress. The mitophagy signaling cascade was upregulated in myocytes from the aged hearts at basal conditions that correlated with elevated oxidative stress and GSSG/GSH ratio. In HF, however, mitophagy was impaired due to improper fusion-fission process which corresponded with decreased MFN2 and DRP1 expression, and led to accumulation of Parkin-rich giant areas in cardiac myocytes. Cell treatment with TAT-MP1^Gly^ peptide, which stabilizes the mitofusin conformation state, enhanced the mitophagy pathway in HF hearts and prevented giant Parkin-rich fusion formation without a significant effect in age matched control hearts. The KB β-hydroxybutyrate elevated mitophagic removal of damaged mitochondria in young and aging myocytes, while it increased the percent of cells with giant Parkin-rich areas in HF. Interestingly, this β-OHB-induced increase in giant Parkin-rich areas was prevented by cell treatment with TAT-MP1^Gly^ peptide, an intervention which stabilizes MFN2 in a conformation that promotes proper fusion, suggesting that β-OHB itself did not induce accumulation of Parkin-rich regions in HF myocytes. These data suggest that, in general, β-OHB increased Parkin translocation to mitochondria in HF myocytes, while it led to the enhanced accumulation of Parkin-rich areas under conditions of impaired fusion-fission. We therefore conclude that β-OHB activates Parkin translocation to mitochondria in all three conditions (young, aged and HF); however, it worsens mitophagy in conditions of improper Mfn2-DRP1 mediated fusion-fission observed in HF.

## Ethics Statement

All animal handling and laboratory procedures were in accordance with the approved protocols of the Institutional Animal Care and Use Committee of the University of California, Davis confirming to the Guide for the Care and Use of Laboratory Animals published by the US National Institute of Health (8th Edition, 2011).

## Author Contributions

All authors contributed substantially to this work. ED designed and supervised this study. PT, LS, CM, and ED performed experiments and analyzed the data. MF provided technical support. ED wrote the manuscript. GD generated and provided MFN2-conformation-modulating peptides. SS and DB edited manuscript and provided financial support. Part of this work has been presented at the Annual Meeting of the Biophysical Society and published in form of the abstract (Miller et al., 2018).

### Conflict of Interest Statement

The authors declare that the research was conducted in the absence of any commercial or financial relationships that could be construed as a potential conflict of interest.

## Abbreviations

β-OHB: β-Hydroxybutyrate
[Ca^2+^]_i_: Cytosolic calcium concentration
[Ca^2+^]_em_: Extramitochondrial free calcium
Drp1: Dynamin-related protein 1
ECC: Excitation-contraction coupling
ETC: Electron transport chain
FCCP: Carbonyl cyanide 4-(trifluoromethoxy)-phenylhydrazone
GRX1-roGFP: The redox-sensitive roGFP fused to human glutaredoxin to sense GSSG
GSH: Reduced glutathione endogenous antioxidant
GSSG: Glutathione disulfide, the oxidized form of glutathione
HF: Heart failure
KBs: Ketone bodies
LC3: Microtubule associated protein 1 light chain 3
LC3-I: Cytosolic form of the microtubule-associated protein 1 light chain 3
LC3-II: Lipidated form of the microtubule-associated protein light chain 3
MFN2: Mitochondrial outer membrane GTPase mitofusin 2
mPTP: Mitochondrial permeability transition pore
ORP1-roGFP: The redox-sensitive roGFP fused with the yeast peroxidase ORP1
Parkin: E3 ubiquitin ligase
PINK1: PTEN-induced putative kinase 1
PHB: Poly-β-hydroxybutyrate
ROS: Reactive oxygen species
TIM: Translocase of the inner mitochondrial membrane
TOM: Translocase of the outer mitochondrial membrane
ΔΨ_m_: Mitochondrial membrane potential.
